# LC‐OCT Features of Confluent and Reticulated Papillomatosis of Gougerot–Carteaud Syndrome

**DOI:** 10.1155/crdm/1417383

**Published:** 2026-06-19

**Authors:** Daniele Omar Traini, Gerardo Palmisano, Andrea Paradisi, Mariarita Vigilante, Alessio Maccari, Alessandro Di Stefani

**Affiliations:** ^1^ Dermatology, Department of Translational Medicine and Surgery, Università Cattolica Del Sacro Cuore, Rome, Italy, unicatt.it; ^2^ Dermatology Unit, Department of Medical and Surgical Sciences, Fondazione Policlinico Universitario a. Gemelli-IRCCS, Rome, Italy; ^3^ Catholic University of the Sacred Heart, Rome, Italy, unicatt.it

## Abstract

Confluent and reticulated papillomatosis (CRP) is a rare acquired keratinization disorder. We report a 29‐year‐old woman with a 3‐year history of mildly pruritic hyperpigmented papules on the back, flanks, abdomen, and groin, coalescing centrally into plaques with a peripheral reticulated pattern. Dermoscopy showed a cerebriform sulci‐and‐gyri‐like network. LC‐OCT revealed a markedly thickened hyperreflective stratum corneum, increased epidermal thickness, and a strongly undulated dermal–epidermal junction; a hyperreflective basal band may also be appreciable if consistently visible on the image set. Histopathology showed orthokeratotic hyperkeratosis, focal acanthosis, papillomatosis, and increased basal pigmentation, confirming the diagnosis of CRP. In this case, LC‐OCT identified in vivo optical features corresponding to key histopathologic criteria of CRP and may support noninvasive clinicopathologic correlation.

## 1. Introduction

Confluent and reticulated papillomatosis (CRP) of Gougerot and Carteaud is a rare, acquired keratinization disorder [1]. Clinically, it typically manifests in young adults with persistent hyperpigmented papules and plaques that are confluent centrally and reticulated peripherally [2]. The lesions are usually asymptomatic or only mildly pruritic and localized on the upper trunk, axillae, upper chest, and upper back. The pathophysiology of CRP remains poorly understood, but an abnormal keratinization in response to *Malassezia furfur* or to *Dietzia papillomatosis*, a Gram‐positive aerobic actinomycete, has been hypothesized [2]. Histopathology reveals epidermal hyperplasia with papillomatosis, compact hyperkeratosis, focal acanthosis, and increased basal melanin [1, 2].

In recent years, noninvasive skin imaging techniques, particularly line‐field confocal optical coherence tomography (LC‐OCT), have emerged as valuable tools for providing high‐resolution in vivo “virtual histology” across a large spectrum of dermatological conditions [3–6]. We describe a case of CRP in a young woman, with direct correlation between LC‐OCT features and histopathologic findings. To our knowledge, this is the first report of LC‐OCT in CRP showing in vivo features corresponding to its key histopathologic criteria.

## 2. Case Presentation

**FIGURE 1:**
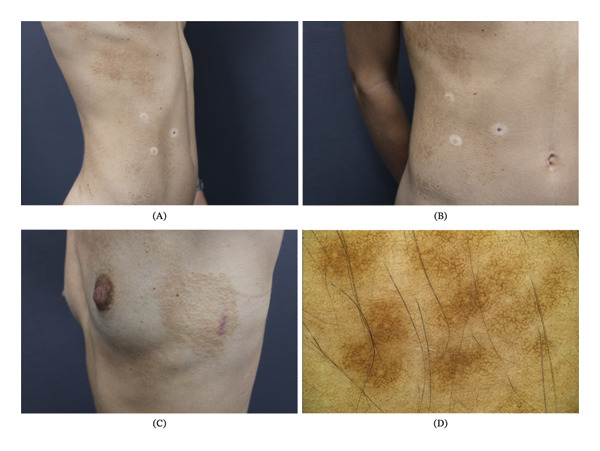
A 29‐year‐old woman presented with a 3‐year history of a mildly pruritic rash involving the back, abdomen, and groin, with numerous brownish papules scattered over the upper and lower back and flanks (A–C). Dermoscopy (magnification: 10x) of an affected area revealed a cerebriform or sulci‐and‐gyri‐like pigment network formed by undulating brown lines (D).

A 29‐year‐old woman presented with a 3‐year history of a mildly pruritic rash involving the back, abdomen, and groin. On examination, there were numerous 3–6 mm brownish papules scattered over the upper and lower back and flanks. Centrally, the papules coalesced into broad brown plaques, while at the periphery a finer reticulated pattern was evident. The patient reported no systemic symptoms. Incidentally, there were 4 Sutton nevi on her trunk that the patient recalled since more than 10 years (Figure 1A–C).

Dermoscopy of an affected area revealed a cerebriform or sulci‐and‐gyri‐like pigment network [7] formed by undulating brown lines, corresponding to papillomatous surface changes (Figure 1D). Wood’s lamp examination revealed no characteristic fluorescence, with only minimal accentuation of the pigmentation.

**FIGURE 2:**
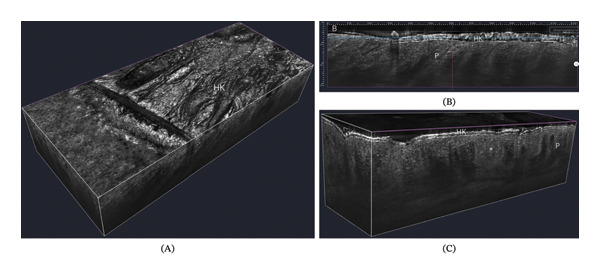
LC‐OCT imaging (A‐C) of an affected site revealed a marked hyperkeratosis (HK) with an irregular and undulating dermal–epidermal junction, with papillomatous projections (P) and focal epidermal thickening (white asterisk).

LC‐OCT imaging of an affected site revealed a markedly thickened hyperreflective stratum corneum, corresponding to orthokeratotic hyperkeratosis. In some acquisitions, it was possible to appreciate focal epidermal thickening, corresponding to acanthosis. The dermal–epidermal junction appeared irregular and undulating, with papillomatous projections (Figure 2). A biopsy from a lesion on the back confirmed the presence of pronounced orthokeratotic hyperkeratosis, papillomatosis, focal acanthosis, and increased basal pigmentation, establishing the diagnosis of CRP.

Therapy with minocycline (50 mg twice/day for 6 weeks) associated with topical tretinoin 0.05% was established, with resolution of the lesions at the three‐month follow up.

## 3. Discussion

CRP remains a rare dermatosis of unknown cause. In most cases, as in our patient, lesions are distributed over the trunk and intertriginous areas, with mild pruritus as the only symptom.

Differential diagnosis includes tinea versicolor, terra firma‐forme dermatosis, Dowling–Degos syndrome, and acanthosis nigricans. Histologically, tinea versicolor is characterized by clusters of short hyphae and round spores within the stratum corneum (“spaghetti and meatballs”) best seen on PAS or silver stains, with minimal epidermal hyperplasia and little to no papillomatosis. In contrast, terra firma‐forme dermatosis shows retained compact orthokeratosis that can be completely removed by vigorous rubbing with isopropyl alcohol; biopsy is typically unnecessary. Dowling–Degos disease presents with reticulate hyperpigmentation of flexures and comedone‐like follicular papules; histology reveals elongated, branching (“antler‐like”) rete ridges with basilar hyperpigmentation and dilated follicular infundibula. Acanthosis nigricans consists of hyperpigmented plaques in intertriginous areas and is often associated with insulin resistance; histology demonstrates papillomatosis with relatively mild hyperkeratosis and limited basal hyperpigmentation, without the cerebriform surface patterning seen in CRP [8].

In this case, LC‐OCT identified in vivo optical features corresponding to the key histopathologic criteria of CRP. LC‐OCT findings were interpreted by two dermatologists with expertise in LC‐OCT, basedon previous literature: the markedly thickened hyperreflective stratum corneum corresponded to orthokeratotic hyperkeratosis, the broadened epidermal band to focal acanthosis, and the strongly undulated dermal–epidermal junction to papillomatosis. These observations suggest that LC‐OCT may support noninvasive diagnosis and clinicopathologic correlation in selected cases.

## Funding

No funding was received for this manuscript.

## Ethics Statement

The patient in this manuscript has given written informed consent for participation in the study and the use of their case details (including photographs) for publication.

## Conflicts of Interest

The authors declare no conflicts of interest.

## Data Availability

The data that support the findings of this study are available on request from the corresponding author. The data are not publicly available due to privacy or ethical restrictions.
